# Dynamic Hormone Control of Stress and Fertility

**DOI:** 10.3389/fphys.2020.598845

**Published:** 2020-11-17

**Authors:** Eder Zavala, Margaritis Voliotis, Tanja Zerenner, Joël Tabak, Jamie J. Walker, Xiao Feng Li, John R. Terry, Stafford L. Lightman, Kevin O'Byrne, Krasimira Tsaneva-Atanasova

**Affiliations:** ^1^Centre for Systems Modelling and Quantitative Biomedicine, University of Birmingham, Birmingham, United Kingdom; ^2^Institute of Metabolism and Systems Research, College of Medical and Dental Sciences, University of Birmingham, Birmingham, United Kingdom; ^3^EPSRC Centre for Predictive Modelling in Healthcare, Living Systems Institute, College of Engineering, Mathematics and Physical Sciences, University of Exeter, Exeter, United Kingdom; ^4^Institute of Biomedical and Clinical Science, College of Medicine and Health, University of Exeter, Exeter, United Kingdom; ^5^Henry Wellcome Laboratory for Integrative Neuroscience and Endocrinology, Translational Health Sciences, Bristol Medical School, University of Bristol, Bristol, United Kingdom; ^6^Department of Women and Children's Health, School of Life Course Sciences, King's College London, London, United Kingdom; ^7^Department of Bioinformatics and Mathematical Modelling, Institute of Biophysics and Biomedical Engineering, Bulgarian Academy of Sciences, Sofia, Bulgaria

**Keywords:** CORT, fertility, GnRH pulse generator, glucocorticoids, hypercortisolism, KNDy network, stress, mathematical model

## Abstract

Neuroendocrine axes display a remarkable diversity of dynamic signaling processes relaying information between the brain, endocrine glands, and peripheral target tissues. These dynamic processes include oscillations, elastic responses to perturbations, and plastic long term changes observed from the cellular to the systems level. While small transient dynamic changes can be considered physiological, larger and longer disruptions are common in pathological scenarios involving more than one neuroendocrine axes, suggesting that a robust control of hormone dynamics would require the coordination of multiple neuroendocrine clocks. The idea of apparently different axes being in fact exquisitely intertwined through neuroendocrine signals can be investigated in the regulation of stress and fertility. The stress response and the reproductive cycle are controlled by the Hypothalamic-Pituitary-Adrenal (HPA) axis and the Hypothalamic-Pituitary-Gonadal (HPG) axis, respectively. Despite the evidence surrounding the effects of stress on fertility, as well as of the reproductive cycle on stress hormone dynamics, there is a limited understanding on how perturbations in one neuroendocrine axis propagate to the other. We hypothesize that the links between stress and fertility can be better understood by considering the HPA and HPG axes as coupled systems. In this manuscript, we investigate neuroendocrine rhythms associated to the stress response and reproduction by mathematically modeling the HPA and HPG axes as a network of interlocked oscillators. We postulate a network architecture based on physiological data and use the model to predict responses to stress perturbations under different hormonal contexts: normal physiological, gonadectomy, hormone replacement with estradiol or corticosterone (CORT), and high excess CORT (hiCORT) similar to hypercortisolism in humans. We validate our model predictions against experiments in rodents, and show how the dynamic responses of these endocrine axes are consistent with our postulated network architecture. Importantly, our model also predicts the conditions that ensure robustness of fertility to stress perturbations, and how chronodisruptions in glucocorticoid hormones can affect the reproductive axis' ability to withstand stress. This insight is key to understand how chronodisruption leads to disease, and to design interventions to restore normal rhythmicity and health.

## 1. Introduction

**Figure 1:**
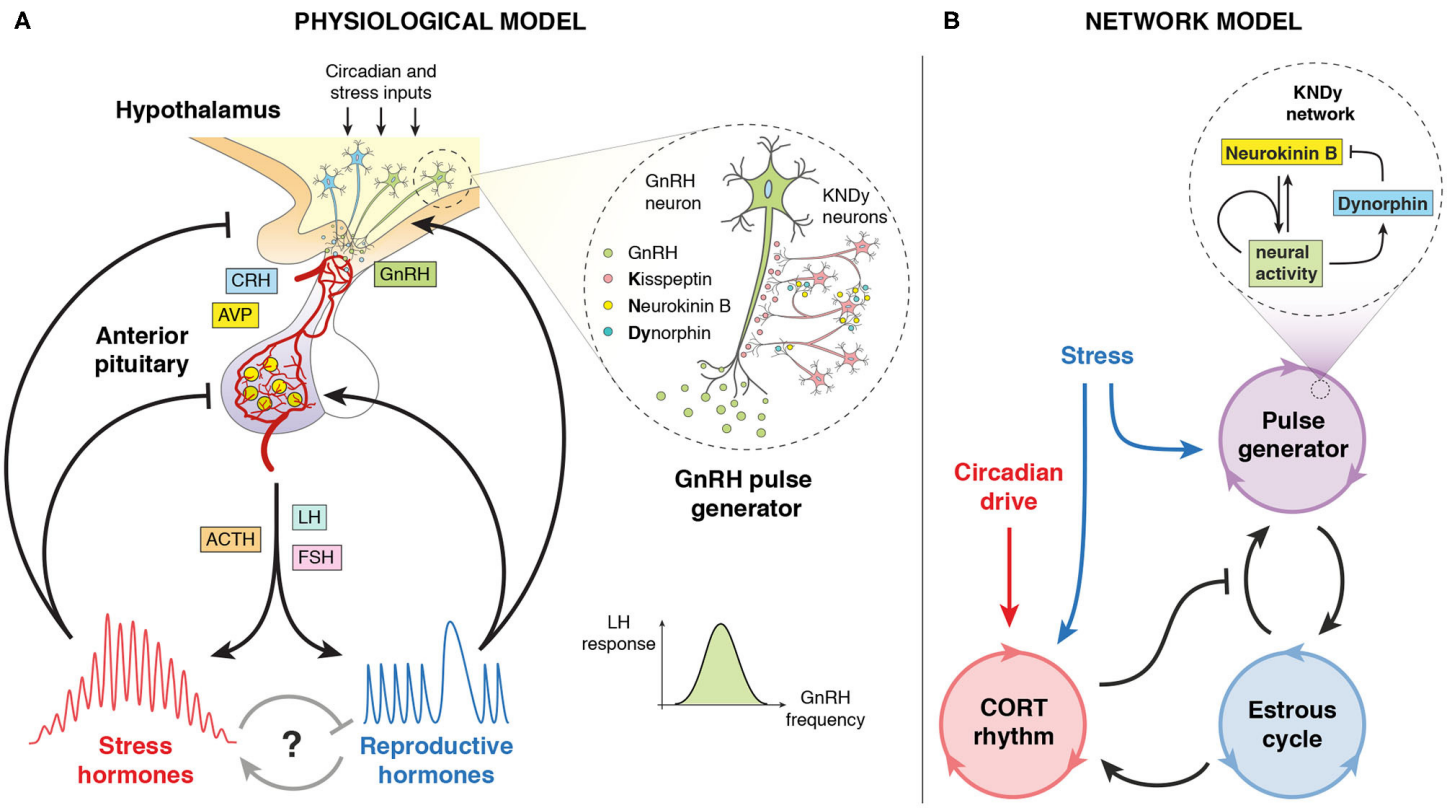
Pictorial representation of the model. **(A)** Physiological model of the stress and reproductive neuroendocrine axes controlling ultradian, circadian, and infradian hormone oscillations. Includes the KNDy neuronal network controlling the GnRH pulse generator. Adapted from Zavala et al. (2019). **(B)** Network model of the systems-level cross-regulation between glucocorticoid (CORT) rhythms, the hypothalamic GnRH pulse generator and the estrous cycle, subject to stress and circadian inputs. Includes a mean-field model of the KNDy network from Voliotis et al. (2019).

A robust dynamic interplay between body rhythms is essential to sustain healthy states. This requires the coordination of several regulatory systems spanning multiple levels of organization, from molecular, to cellular, to the whole organism. Network physiology approaches employ analytical tools, such as mathematical modeling to investigate the interactions between organs and their integration into physiological systems. Neuroendocrine axes are the perfect example of such interlocked-regulatory systems controlling body rhythms, with the brain decoding circadian and stress inputs as well as integrating feedback signals from endocrine organs. The hypothalamic-pituitary-adrenal (HPA) axis and the hypothalamic-pituitary-gonadal (HPG) axis are the major neuroendocrine systems underpinning stress and fertility, respectively. These axes control a range of hormonal and neural activity rhythms exhibiting ultradian (<24 h), circadian (~24 h) and infradian (>24 h) periodicity (Walker J. et al., 2010), as well as responses to environmental, biological and behavioral perturbations. For example, the HPA axis uses feedback control to regulate stress responses while sustaining ultradian and circadian glucocorticoid (CORT) rhythms (Walker J. J. et al., 2010; Spiga et al., 2017). On the other hand, the HPG axis controls infradian oscillations of reproductive hormones secreted in response to changes in the ultradian frequency of gonadotropin-releasing hormone (GnRH). GnRH secretion is controlled by a hypothalamic pulse generator (PG) (Voliotis et al., 2019), which is in turn modulated by gonadal hormones (Figure 1A). Mathematical modeling has significantly contributed to our understanding of this rhythmic behavior (Zavala et al., 2019; Clément et al., 2020), as well as the ability of these systems to respond to perturbations (Spiga et al., 2017). For instance, a mathematical model of rhythmic HPA axis activity has been introduced in Walker J. J. et al. (2010). In this model, ultradian CORT pulsatility is generated by a pituitary-adrenal feedback loop. The model predicts that, in contrast to the reproductive axis, in the stress axis the hypothalamus only needs to provide circadian amplitude modulation of ultradian CORT pulses to explain experimental observations (Walker et al., 2012). Regarding the HPG axis, a combination of mathematical modeling and experimental physiology has shown how the hypothalamic kisspeptin neuronal network generates and sustains pulsatile LH secretion (Voliotis et al., 2019). More recently, a generalized integrate and fire model has been postulated as a simple mechanism to generate a range of rhythmic neuroendocrine signals (Churilov et al., 2020).

Most of the evidence on the dynamic interactions between the HPA and HPG axes comes from animal studies (Acevedo-Rodriguez et al., 2018; McCosh et al., 2019). For example, experiments in ovariectomized rats show a reduction in circadian levels of CORT, which is restored to physiological levels with estradiol administration (Seale et al., 2004a,b, 2005a,b). Data from rodents also shows how physiological and psychosocial stressors can temporarily disrupt GnRH pulse generator activity. These stressors range from isolation and restrain, to insulin induced hypoglycemia and high exogenous CORT, with evidence suggesting the involvement of kisspeptin neuron activity (Li X. F. et al., 2004; Luo et al., 2016; Yang et al., 2017; Ayrout et al., 2019; Kreisman et al., 2019). Further studies in macaque have shed light on the sensitivity and resilience of the reproductive axis to stress signals (Herod et al., 2011a,b). Human studies have also highlighted the profound effect of glucocorticoid excess on the menstrual cycle (Ding et al., 1988; Suh et al., 1988; Saketos et al., 1993; Crofford et al., 1999). However, there is still a limited understanding of whether and how the HPA and HPG axes coordinate their hormone rhythms, how perturbations to one axis impact upon the other, what makes their dynamics robust to such perturbations, and in what circumstances chrono-disruptions can lead to disease.

In this manuscript, we investigate the dynamic control of stress and fertility by means of a mathematical model that accounts for the complex interactions between the HPA and HPG axes. First, we postulate that these neuroendocrine systems behave as a network of coupled oscillators that coordinate ultradian, circadian and infradian rhythms, and validate the model predictions against published physiological observations in female rodents. Second, we consider the evidence on stress-induced suppression of GnRH pulse generator activity dependent of estradiol, and use the model to understand the role of estradiol-dependent effects on the HPA axis (Seale et al., 2004b; Phumsatitpong and Moenter, 2018). We also simulate the simultaneous effect of exogenous estradiol and glucocorticoids on the dynamics of the GnRH pulse generator (Kreisman et al., 2019). Third, we use the model to explore how perturbations, such as stressors and chronic changes in gonadal steroids and glucocorticoid levels can disrupt normal rhythmicity and lead to dysregulations that propagate from one neuroendocrine system to the other. To do so, we consider typical restraint stress signals (Li X. et al., 2004) to brain regions that are connected to the hypothalamus, thus affecting both the HPA and HPG axes (Li et al., 2005; Herod et al., 2011b). Importantly, our model considers the signaling role of regulatory neuropeptides (e.g., Neurokinin-B and Dynorphin) within the KNDy neural network in stress-induced suppression of the GnRH pulse generator (Lehman et al., 2010; Grachev et al., 2014; Voliotis et al., 2019), which has implications for our understanding of how stress signals are decoded by the reproductive axis. Lastly, we predict an increase of the estrous cycle length under hiCORT and discuss how our model can help understand the mechanisms allowing robust control of ovulation despite the effect of stressors.

## 2. Model and Methods

### 2.1. Mathematical Modeling

We focus on the systems-level outputs and cross-regulation of the stress and reproductive axes, which in turn we model as a network of coupled oscillators (Figure 1B). We modeled this through a system of Ordinary Differential Equations (ODEs), where each oscillator represents an aspect of neuroendocrine rhythmic activity that can be characterized by a phase φ, a frequency ω, and an amplitude *A*. Our model consists of a master circadian oscillator in the hypothalamus, a glucocorticoid (CORT) oscillator with ultradian rhythmicity driven by the circadian oscillator, a pulse generator oscillator governed by the Kisspeptin, Neurokinin B, Dynorphin (KNDy) network regulating pulses of GnRH secretion, and an oscillator representing the estrous cycle. The equations for these oscillators are listed below, with coupling functions, parameter values, and further details of the model development described in the Supplementary Material.

#### 2.1.1. Circadian Cycle

A fixed period hypothalamic oscillator to control the circadian rhythm of CORT:

(1) $$\begin{array}{r} \frac{d}{dt}\varphi_{H}=\omega_{H_{0}}, \end{array}$$

where φ_*H*_ is the hypothalamic phase and ω_*H*_0__ is the natural frequency of the hypothalamic circadian drive.

#### 2.1.2. CORT Oscillator

Accounts for CORT ultradian oscillations originating from the pituitary-adrenal feedback loop (Walker J. J. et al., 2010). Its dynamics can be affected by stressors, exogenous CORT, and the estrous cycle. The phase φ_*C*_ is given by:

(2) $$\begin{array}{r} \frac{d}{dt}\varphi_{C}=\omega_{C_{0}}-\alpha s\left(\varphi_{H}\right), \end{array}$$

where ω_*C*_0__ is the natural frequency of CORT ultradian oscillations, *s*(φ_*H*_) is a function accounting for a transient acute stressor (equal to zero in the absence of stress), and α is a scaling factor accounting for how strongly such stressor temporarily disrupts CORT ultradian rhythmicity. The amplitude *A*_*C*_ is given by:

(3) $$\begin{array}{r} \frac{d}{dt}A_{C}=f_{H}\left(\varphi_{H}\right)\frac{{A_{E}}^{n}}{{A_{E}}^{n}+{K_{E}}^{n}}-A_{C}+s\left(\varphi_{H}\right), \end{array}$$

where *f*_*H*_(φ_*H*_) is a function representing hypothalamic circadian modulation. *A*_*E*_ is the amplitude of the estrous cycle (representative of the level of sex steroids) which modulates *A*_*C*_ through a Hill type function with coefficient *n* and half-maximum constant *K*_*E*_.

#### 2.1.3. Pulse Generator

Accounts for the activity of the GnRH pulse generator. Its frequency is modulated by stressors, CORT levels, and the activity of the KNDy network (Voliotis et al., 2019), which is in turn influenced by the phase of the estrous cycle. The phase φ_*PG*_ is given by:

(4) $$\begin{array}{r} \frac{d}{dt}\varphi_{PG}=\omega_{PG}, \end{array}$$

where ω_*PG*_ denotes the varying frequency of the pulse generator. This is given by:

(5) $$\begin{array}{r} \frac{d}{dt}\omega_{PG}=\omega_{PG_{m}}f_{K}\left(N,D,\tilde{C}\right)-\omega_{PG}, \end{array}$$

where ω_*PG*_*m*__ is the maximum frequency of the pulse generator and $f_{K}\left(N,D,\tilde{C}\right)$ is a function accounting for the regulation from the KNDy network and CORT. Equations for the excitatory (*N*; e.g., Neurokinin B and glutamate) and inhibitory (*D*; e.g., Dynorphin) signals regulating the frequency of the KNDy network, and the slow genomic CORT effects ($\tilde{C}$) are given in the Supplementary Material.

#### 2.1.4. Estrous Cycle

Accounts for the activity of the reproductive cycle. The phase φ_*E*_ is given by:

(6) $$\begin{array}{r} \frac{d}{dt}\varphi_{E}=\omega_{E_{m}}f_{PG}\left(\omega_{PG}\right), \end{array}$$

where *f*_*PG*_(ω_*PG*_) is a function accounting for the effects of the pulse generator and ω_*E*_*m*__ is the maximum frequency of the estrous cycle. The amplitude *A*_*E*_ is given by:

(7) $$\begin{array}{r} \frac{d}{dt}A_{E}=\varepsilon +\beta f_{E}\left(\varphi_{E}\right)-A_{E}, \end{array}$$

where ε is the basal activity of the estrous cycle, *f*_*E*_(φ_*E*_) is a function representing the effects of the estrous cycle, and β is a scaling factor accounting for the strength of such effects.

### 2.2. Computer Simulations and Parameter Estimation

To simplify our analysis, CORT oscillations were normalized to the maximum levels observed in physiological conditions. That is, the CORT amplitude, which is modulated by the circadian drive, spans the range between 0 and 1 unless stressors or exogenous CORT act upon it. Similarly, the activity of the PG was represented by normalized oscillations, with a frequency that changes periodically according to the different stages of the estrous cycle.

For the scenarios in sections 3.2 and 3.3, we model the estrous cycle regulation of its amplitude and its effects on the KNDy network through a skewed sinusoidal function of the phase φ_*E*_. This is given by:

(8) $$f_{E}(\varphi_{E})=\{\begin{array}{ll} \sin^{2}\left(\varphi_{E}-\sigma \sin^{2}(\varphi_{E})\right), & \text{Normal physiological}\\ 0.99, & \text{OVX}\\ 0.2, & {\text{OVX}+\text{E}}_{2} \end{array}$$

where σ is the skewness of the estrous cycle. We fix *f*_*E*_(φ_*E*_) to a constant value to simulate the OVX and OVX + E_2_ scenarios (Seale et al., 2004b; Kreisman et al., 2019). Note that in those cases, parameters ε and β in the equation for *A*_*E*_ also need to change as indicated in Supplementary Table 3 to reflect estrous activity expected in the diestrus and proestrus phase.

The model equations were numerically solved and analyzed in MATLAB R2020a using ode45 routines. Details of the mathematical model development and parameter values are described in the Supplementary Material. The model parameters were estimated from the literature where available and manually calibrated to reproduce experimental observations of CORT and reproductive rhythms in rodents.

No new data involving animal or human subjects is presented in this paper.

## 3. Results

### 3.1. Normal Physiological HPA and HPG Rhythms

**Figure 2:**
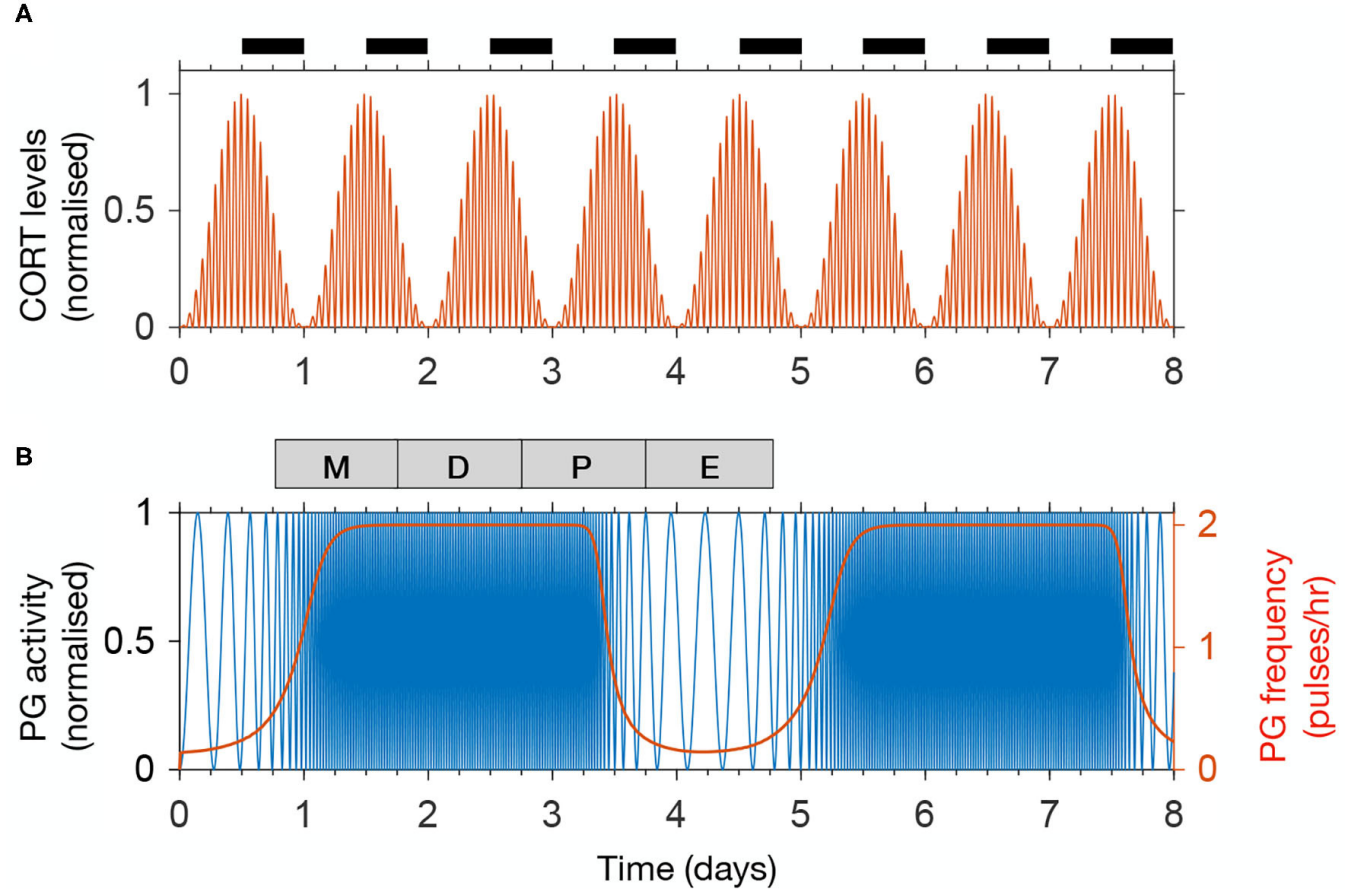
The model reproduces physiological rhythms in the HPA and HPG axis. **(A)** Normalized CORT levels as a function of time. The light-dark cycle is represented with intermittent black bars on the top. **(B)** Normalized pulse generator activity (blue) and pulse generator frequency (red) as a function of time. The phases of the estrous cycle are marked on the top: estrus (E); metestrus (M); diestrus (D); and proestrus (P).

We calibrate the model parameters to reproduce physiological HPA and HPG rhythms observed in rats (Walker J. et al., 2010). Accordingly, our model simulates CORT oscillations with a 75 min period, while the amplitude of these ultradian pulses is modulated in a circadian manner, reaching a maximum at the start of the dark period (Figure 2A). Furthermore, one full estrous cycle lasts ~4 days, matching the average cycle length measured in rats (McClintock, 1984). A recent study using fiber photometry calcium imaging from arcuate kisspeptin neurons in mice revealed the dynamic modulation of GnRH pulse frequency along the estrous cycle (McQuillan et al., 2019). Following these findings, the activity of the PG in the model remains inhibited (below 1 pulse/h) during the post-ovulatory, estrous phase, rises steeply at the start of metestrus, and levels off at 2 pulses/h for the rest of the cycle (Figure 2B).

### 3.2. Recovery of CORT Dynamics Following Ovariectomy

**Figure 3:**
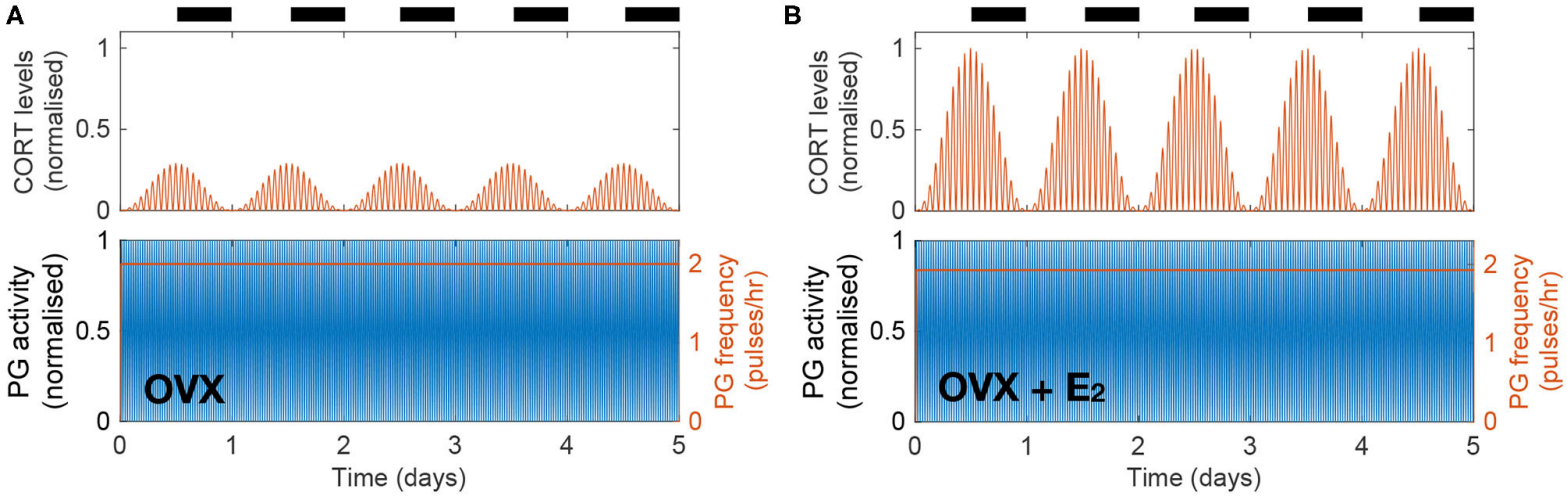
The model explains how E_2_ replacement recovers physiological CORT levels in OVX rats. **(A)** Simulated OVX reduced CORT oscillations down to ~30% of the maximum physiological levels while keeping a constant high PG activity. **(B)** Simulated OVX + E_2_ recovered CORT oscillations to physiological levels while keeping a constant high PG activity.

Previous findings suggest that gonadal steroids are integral to the increased CORT levels seen in females compared to males. This has been demonstrated by showing the effects of estrogen replacement in recovering physiological CORT levels following ovariectomy in rats (Seale et al., 2004b). We investigate the dynamic effects of these hormones by simulating the inhibition of HPA axis activity resulting from ovariectomy (OVX) and its restitution following 17β-estradiol (E_2_) replacement. In the model, this is achieved by replacing the influx term in the right hand side of Equation (7) by a constant term representing a drop in E_2_ levels following OVX (causing *A*_*E*_ to drop down to a constant level of 2% from the estrous peak) and by replacing the periodic sensitivity of the KNDy network to the estrous phase by a constant low value (Supplementary Material). The model predicts a drop in CORT levels down to ~30% from its physiological value without loss of circadian or ultradian CORT rhythmicity while keeping the PG frequency at a high constant value of 2 pulses/h (Figure 3A). We then simulated the effects of an E_2_ pellet on OVX rats by increasing the constant value of the influx term in the right hand side of Equation (7) (98% from physiological *A*_*E*_) and increasing the sensitivity of the KNDy network to the estrous phase (φ_*E*_) by a constant value (Supplementary Material). In agreement with Seale et al. (2004b), the model predicts recovery of physiological CORT levels without loss of circadian or ultradian CORT rhythmicity while marginally reducing the PG frequency just below 2 pulses/h (Figure 3B).

### 3.3. Estradiol-Mediated Inhibition of HPG Dynamics by High CORT Doses

In a recent study, Kreisman et al. (2019) investigated the effect of chronic CORT administration on LH pulsatility and demonstrated the importance of gonadal steroid hormones in mediating the inhibitory effect of CORT on the HPG axis. The study showed that a pellet delivering a high dose of CORT over 48 h in OVX mice has no effect on LH pulsatility, whereas a significant reduction of LH pulse frequency is observed in OVX animals treated with a 17β-estradiol silastic implant (OVX + E_2_). In our model, we accounted for the OVX and OVX + E_2_ scenarios as described in the previous section, while the constantly high CORT levels were achieved by replacing the effective CORT levels modulating the KNDy network by a constant high value estimated from Kreisman et al. (2019) (see Supplementary Material).

**Figure 4:**
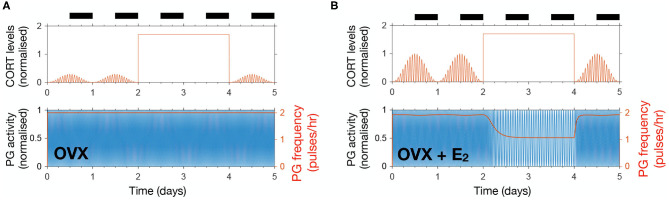
The model reproduces estradiol-mediated inhibition of PG activity following high doses of CORT. **(A)** High exogenous CORT over 48 h does not affect the PG dynamics in OVX mice. **(B)** In the presence of estradiol, high CORT doses temporarily reduce PG activity in OVX mice.

Figure 4 illustrates the differential effect of chronically elevated CORT levels on the GnRH pulse generator frequency in OVX vs. OVX + E_2_ animals. In the case of OVX animals, elevated CORT levels do not alter the frequency of the pulse generator, whereas in OVX animals treated with estradiol the frequency is halved for as long as CORT levels are elevated. This effect is linked to the modulation of the GnRH pulse generator by gonadal steroids, which sensitize the system to inhibitory signals, such as CORT or acute stressors as we show below (Figure 4B).

### 3.4. Acute Stress Effects on the HPA and HPG Axes Depend on the Estrous Cycle Phase

To study the effect of acute stress on the dynamics of the HPA and HPG axes, we extend the model to include transient stress-related neuronal inputs affecting both axes (Yang et al., 2017). In our model, we account for these transient inputs by simulating a 2 h square pulse of amplitude 1, equivalent to a restraint stressor causing a CORT increase from its circadian nadir up to its circadian peak (Kitchener et al., 2004). The stressor affects the phase and amplitude of the CORT rhythm (function *s*(φ_*H*_) in Equations 2 and 3) as well as the frequency of the GnRH pulse generator (function $f_{K}\left(N,D,\tilde{C}\right)$ in Equation 5 and Supplementary Material).

**Figure 5:**
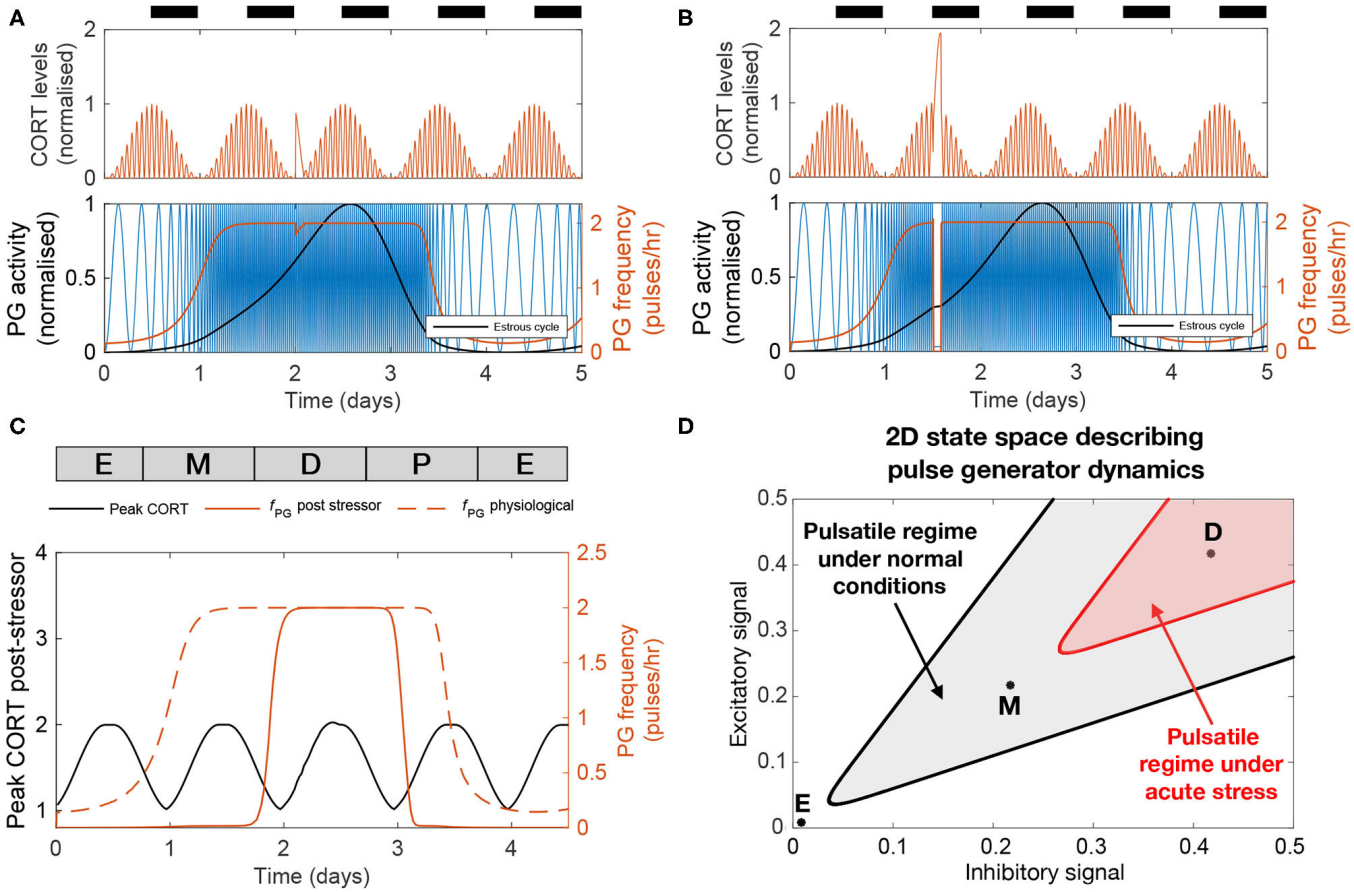
The effect of acute stress on the dynamics of the HPA and HPG axes. **(A,B)** CORT levels and PG activity in response to a transient (2 h long) stressor initiated at two different times. **(C)** Peak CORT levels (black line) and mean PG frequency (continuous red line) elicited by a 2 h long acute stressor as a function of the time at which the stressor arrives during the estrous cycle. The PG frequency without any stress perturbation is shown for comparison (dashed red line). **(D)** State space diagram describing the effect of acute stress on the dynamics of the pulse generator. Points mark different stages along the estrus cycle: estrus midpoint (E); metestrus midpoint (M); and diestrus midpoint (D). The shaded gray area denotes the region of the state space corresponding to frequencies above 1 pulse/h under normal physiological conditions. Acute stress shrinks this region (red shaded area), but the dynamics of the pulse generator maintains robustness to perturbations during the diestrus phase.

Figures 5A,B illustrate the effect that 2 h of stress activation has on the dynamics of the HPA and HPG axes when applied at different times along the cycle. Both CORT and GnRH pulse generator responses are dependent on the timing of the input pulse (Figure 5C). The amplitude of the CORT response shows a circadian dependency with stressors delivered during the circadian peak eliciting a stronger response. The GnRH pulse generator frequency response to acute stressors depends on the phase of the estrous cycle. In particular, the frequency of the pulse generator appears most sensitive to stressors during estrus to early diestrus phases, with little or no effect during the mid-cycle phase. This differential effect of acute stress on the frequency of the GnRH pulse generator activity highlights the cycle dependent modulation of the pulse generator dynamics, which makes the pulse generator more robust to perturbations in the diestrus and proestrus phases (Figure 5D).

### 3.5. CORT Excess Increases the Length of the Estrous Cycle and Modulates Responses to Acute Stressors

**Figure 6:**
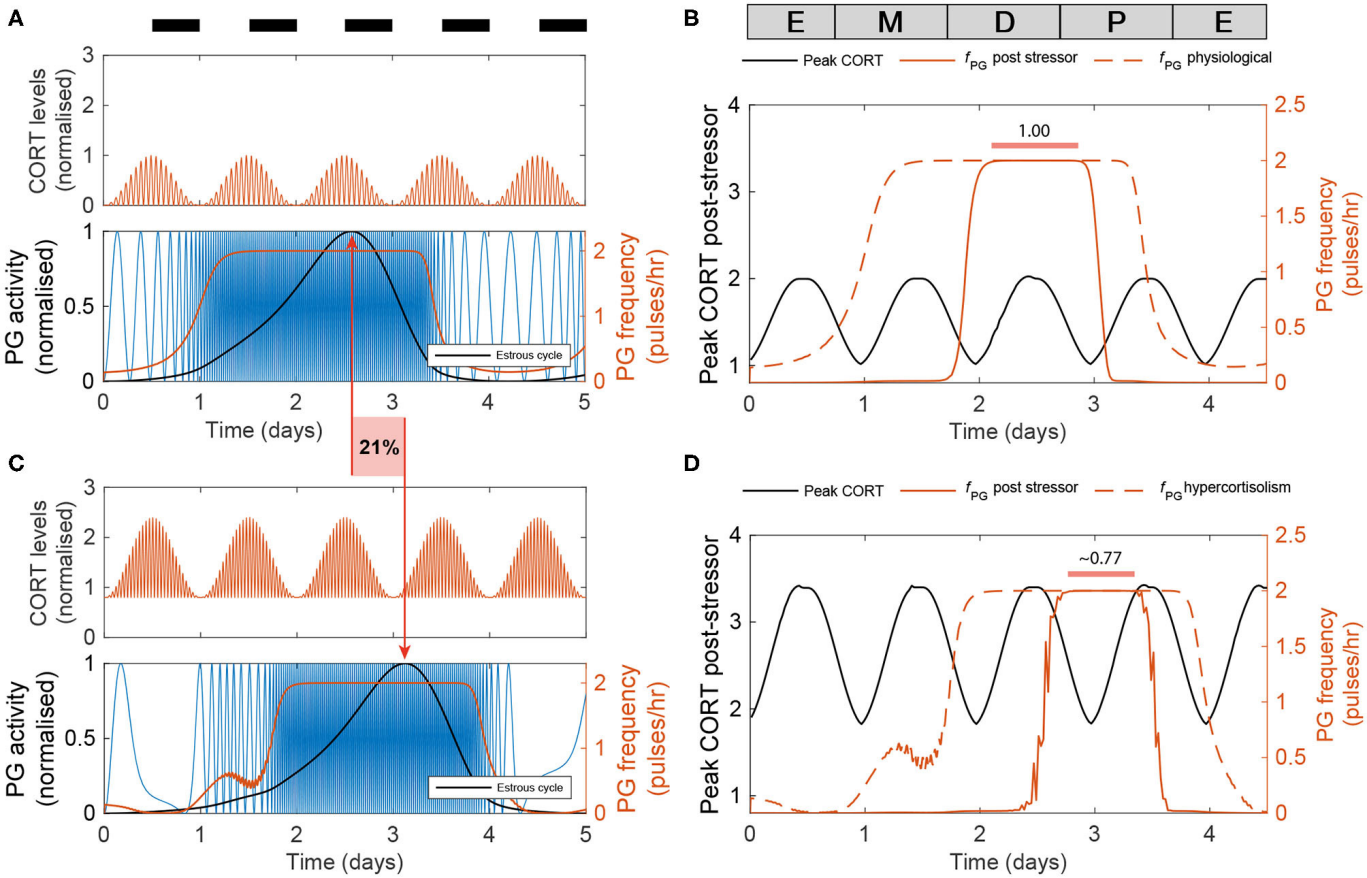
Enduring and transient dynamic changes under hiCORT. **(A)** CORT and PG rhythms in physiological conditions. **(B)** Mean PG frequency and maximum CORT levels elicited by 2 h long hypothalamic stressors arriving at different times across the estrous cycle. **(C)** Under hiCORT, the range of CORT levels is increased and the estrous cycle peak is delayed by ~21% compared to physiological values. **(D)** Mean PG frequency and maximum CORT elicited by 2 h long stressors under hiCORT. In this scenario, the period in which PG activity remains unchanged by stressors starts about half a day later and is shortened compared to normal physiological conditions.

Last, we used the model to predict the effects of high excess CORT (hiCORT) —mimicking levels expected to be observed in people with hypercortisolism—on the estrous cycle. To do this, we considered the increase in baseline and maximum CORT amplitude with respect to physiological levels in humans (Vagnucci, 1979) and implemented the equivalent increase ratios for our simulations of CORT dynamics in rodents (Supplementary Material). Evidence from high frequency sampling in humans shows hypercortisolism is associated with a reduction in the ultradian period of CORT oscillations (Van Aken et al., 2005). Accordingly, we also adjusted this parameter when modeling hiCORT, while keeping circadian oscillations and all other parameters unchanged. Our simulations predict an increase in the period of the estrous cycle from a physiological value of T_*phys*_ = 4.2 days up to T_*hiC*_ = 5.1 days under hiCORT, which is equivalent to a ~21% increase in the estrous cycle length (Figures 6A,C).

We then used the model to investigate the transient changes in the GnRH pulse generator frequency and CORT amplitude elicited by exogenous acute stressors under physiological conditions and hiCORT. In particular, we look at the effects of the timing of stressors within the estrous cycle. To do this, we calculated frequency and amplitude response curves by simulating a 2 h long stressor elicited at different stages across the estrous cycle using 30 min time steps. In the physiological scenario (Figure 6B), the model predicts that acute stressors suppress PG activity during most of the estrous cycle except during the diestrus and early proestrus phases. These stressors also elicit an increase in peak CORT levels to a range between 1 and 2. While the model under the hiCORT scenario predicts a similar behavior, the region where PG activity remains unaffected by acute stressors is reduced and delayed by about half a day compared to its physiological counterpart (Figure 6D and Supplementary Figure 2). This is not surprising considering that the model also predicts that hiCORT prolongs the estrous cycle. Regarding the CORT response to stressors under hiCORT, our model predicts a ~2 to ~3.5 increase in CORT levels compared to the normal physiological scenario. This is due to a compounded effect of CORT surges over an excess CORT baseline.

## 4. Discussion

We developed and studied a mathematical model that integrates components of the stress and reproductive axes at different spatial and temporal scales, from the molecular intricacies of the KNDy network, to GnRH and CORT oscillations, up to the estrous cycle (Figure 1A). Previous mathematical models of the HPA and HPG axes either focus on a specific process within an axis, or consider them as a whole, but isolated from each other (Walker J. J. et al., 2010; Spiga et al., 2017; Voliotis et al., 2019; Clément et al., 2020). In contrast, our model integrates these neuroendocrine axes by considering the complex interactions between them as a network of interlocked oscillators, hence enabling us to integrate different physiological observations and experiments into a single coherent theoretical framework and study the effect of transient perturbations on the overall dynamics. In particular, our model postulated a network architecture (Figure 1B) that reflects physiological observations of ultradian and circadian CORT rhythms, as well as ultradian and infradian rhythms of the GnRH pulse generator (Figure 2). The model reproduced the effects of ovarian hormone removal (OVX) and restitution (E_2_) on the HPA and HPG axes dynamics, both under physiological conditions (Seale et al., 2004b) (Figure 3) and under exogenous CORT excess (Kreisman et al., 2019) (Figure 4).

In addition to these slow timescale perturbations, we also investigated the fast timescale perturbations elicited by acute stressors. Our model predicted that exogenous stress perturbations not only cause transient increases in CORT levels, but also transiently inhibit GnRH pulse generator activity with the magnitude of this inhibition being dependent on the estrous and circadian phases (Figure 5). This has important implications about understanding how the timing of a stressor affects its ability to temporarily suppress the GnRH/LH ovulatory surge. According to our model, the pulse generator activity is robust to stress perturbations arriving between the diestrus and early proestrus stage, but is fragile to stressors arriving at estrus and metestrus stages (Figure 5C). Uncovering the origin of this robustness is beyond the scope of our phenomenological model, but we can speculate that molecular mechanisms ensure the resilience of the reproductive cycle during the key stages leading to ovulation. While our model suggests that the GnRH/LH surge should be delayed under frequent exposure to stressors, if the exposure occurs too close to the proestrus stage then these resilience molecular mechanisms ensure the surge continues as normal and triggers ovulation (Wagenmaker et al., 2010; Wagenmaker and Moenter, 2017).

We also used the model to investigate the potential detrimental effects on fertility elicited by chronic hypercortisolism. Our model predicted that hiCORT delays the increase in activity of the GnRH pulse generator, effectively prolonging the estrous cycle (Figures 6A,C). While evidence suggests that HPA axis hyperactivity—and specifically, increased circulating glucocorticoids—are unlikely to be the sole mechanism behind stress-induced reproductive dysfunction (Herod et al., 2011a), our simulations show the cycle length depends on the GnRH pulse generator's sensitivity to CORT (Supplementary Figure 1A). Thus, our model provides insight into how for example a hyper-sensitized HPG axis may explain amenorrhea secondary to high serum cortisol levels (Ding et al., 1988; Suh et al., 1988; Saketos et al., 1993). Interestingly, our model predicted that a period of robustness of the GnRH pulse generator in the presence of stressors is preserved under hiCORT, albeit the robust period occurs about half a day later in the cycle and is shorter in duration. Our model simulations of pulse generator activity suggest that prolonging the estrous cycle as predicted under hiCORT arises from a combination of longer estrus and metestrus stages while diestrus and proestrus stages are shortened (Figures 6B,D).

Our model considers essential features of HPA and HPG axes oscillators in a phenomenological way. This approach facilitates the simulation of a range of physio-pathological scenarios, but inevitably imposes certain limitations. In contrast to mechanistic models where parameters are often linked to chemical kinetic rates, the parameters in our model represent natural and maximum frequencies, phase relationships, as well as the coupling strengths between oscillators and sensitivities to perturbations. While our phenomenological approach limits the ability of the model to support discovery of specific molecular mechanisms, it can be used to suggest experiments that explore systems level properties involving both neuroendocrine axes. For example, evidence shows that in addition to exhibiting circadian and ultradian fluctuations, CORT levels also change across the estrous cycle, with maximum levels around the diestrus and proestrus phase (Carey et al., 1995; Atkinson and Waddell, 1997; Pilorz et al., 2009). While our model lacks the level of detail to describe the molecular mechanisms that underpin estrous changes on CORT, it does suggest this is mediated by a regulatory link from the estrous oscillator to the CORT oscillator, thus inferring that ovarian steroids may be the culprit of estrous regulation of CORT instead of the hypothalamic GnRH pulse generator (Figure 1). In our model, we only explored the scenario where the strength of this regulatory link allows for strong perturbations (e.g., stressors, OVX, E_2_) in the estrous oscillator to have an impact on the CORT dynamics, but not from milder estrous regulation of CORT levels (Supplementary Figure 1). We speculate that combining mechanistic modeling with experimental physiology to investigate the effects of estradiol and progesterone on CORT may uncover the origins of its estrous cycle modulation. The experiments could test the dosing effect, timing, and combined sensitivity of gonadal steroids on circadian CORT levels across the estrous cycle. The mechanistic model could in turn help understand the robustness of such regulatory mechanism to perturbations (Wagenmaker et al., 2010), and predict the scenarios in which chronodisruptions would lead to disease.

We believe that the first generation mathematical model presented here could be used to inform further investigations into the timing of stress perturbations in reproductive health, including dysregulations induced by strenuous exercise (Ding et al., 1988), mood disorders (Young and Korszun, 2002), as well as clinical interventions, such as *in vitro* fertilization (Massey et al., 2016). Our model is the latest of a class of mathematical models that can support or replace animal studies in endocrinology (Zavala et al., 2019). It can also help design new studies that reduce the number of experiments necessary to refine our understanding of the HPA and HPG axis. Furthermore, computational models like ours can be used to contextualize the results of clinical studies where experimentation is not possible. This can be done in combination with a range of tools from network physiology and machine learning that consider the dynamic links between coupled body rhythms, such as body temperature and sleep (Bashan et al., 2012; Bartsch et al., 2015; Ivanov et al., 2016). We anticipate that healthcare technologies, such as wearable devices and smartphone apps collecting vast amounts of data on body rhythms, together with computer algorithms characterizing inter-individual variability, will help refine and personalize neuroendocrinological models (Kim et al., 2020; Li et al., 2020; Wang et al., 2020).

## Data Availability Statement

The original contributions presented in the study are included in the article/Supplementary Material, further inquiries can be directed to the corresponding author/s.

## Author Contributions

EZ, MV, XL, JRT, SL, KO'B, and KT-A conceived and designed the study. EZ, MV, TZ, JT, JW, and KT-A developed the mathematical model. EZ, MV, and TZ performed the analytical calculations and computer simulations, and wrote the manuscript with support of all authors.

## Conflict of Interest

The authors declare that the research was conducted in the absence of any commercial or financial relationships that could be construed as a potential conflict of interest.
